# Unlocking Klockmannite: Formation of Colloidal Quasi‐2D CuSe Nanocrystals and Photo‐Physical Properties Arising From Crystal Anisotropy

**DOI:** 10.1002/smll.202512836

**Published:** 2026-01-29

**Authors:** Urvi Parekh, Nadiia Didukh, Samira Dabelstein, Ronja Piehler, Eugen Klein, Jivesh Kaushal, Tobias Korn, Stefan Lochbrunner, Christian Klinke, Stefan Scheel, Rostyslav Lesyuk

**Affiliations:** ^1^ Institute of Physics University of Rostock Rostock Germany; ^2^ Department “Life, Light & Matter” University of Rostock Rostock Germany; ^3^ Pidstryhach Institute for Applied Problems of Mechanics and Mathematics of NAS of Ukraine Lviv Ukraine

**Keywords:** copper selenide, klockmannite, plasmon, anisotropy, coherent phonons, hyperbolic, Raman spectrum

## Abstract

Copper selenide is an exceptional quasi‐layered monolithic material that exhibits both semiconducting and metallic properties in adjacent visible and near‐infrared (NIR) spectral ranges. Here we introduce a thiol‐free colloidal synthesis for generating quasi‐2D klockmannite copper selenide nanocrystals via hot injection method, achieving shape control by tuning the injection temperature and precursor concentrations without any additional ligands. This approach produces large klockmannite nanosheets with lateral sizes from 200 nm to several micrometers, as well as uniform triangular nanoplatelets with sizes of 12–25 nm that are monocrystalline and display strong NIR plasmonic absorption. The spectral features of the anisotropic klockmannite phase in the NIR have been analyzed using complex‐scaled discrete dipole approximation (CSDDA) calculations, which reveal pronounced optical anisotropy and the emergence of hyperbolic regime. The combined effect of propagating and evanescent fields is regarded as the underlying reason of such modes in the hyperbolic domain. Finally, the ultrafast photophysical behavior of the material in klockmannite phase is examined, including hot‐hole cooling, trapping, and coherent phonons generation. Our findings emphasize the important role of the intrinsic crystal anisotropy in governing the physical properties of nanoscale klockmannite.

## Graphical abstract

Thiol‐free hot injection approach yields quasi‐2D klockmannite CuSe nanocrystals, specifically nanosheets and uniform triangular nanoplatelets. This study reconsiders the klockmannite from the point of view of crystal anisotropy and demonstrates its effect on plasmonic response in the near‐infrared spectral region with the theoretical emergence of hyperbolicity and mixing of propagating and evanescent fields in triangular nanocrystals.

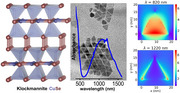

## 1 Introduction

In recent years, metal chalcogenides have garnered strong attention as inorganic semiconductors due to their potential use in solar cells, photodetectors, thermoelectric converters, photocatalysts, flexible electronics and a wide range of other optoelectronic applications [1, 2, 3, 4, 5, 6, 7]. The main constituents of groups II‐VI, IV‐VI, and III‐V metal chalcogenides often contain toxic or rare elements. Materials including Cu_2_S, CuInS_2_, CuInSe_2_, CIGS and CZTS have long been used as thin‐film absorbers [8, 9], and their colloidal nanocrystals [10] (NCs) have more recently enabled solution‐processable devices, conductive films, and photothermal or photocatalytic applications in various media [11, 12, 13, 14, 15, 16, 17]. Also, copper selenides (Cu_x_Se) are among such significant metal chalcogenides, comprising a variety of stoichiometric and non‐stoichiometric phases, including cubic (Cu_2_Se or Cu_2‐x_Se), monoclinic (Cu_2_Se), tetragonal (Cu_3_Se_2_), hexagonal klockmannite (CuSe or Cu_0.87_Se), orthorhombic (Cu_5_Se_4_ or CuSe) and marcasite (CuSe_2_) [18] phases. All these phases exhibit p‐type semiconductor behavior due to the presence of copper vacancies in their crystal lattice with bandgaps covering the range of 1.2 to 2.4 eV [19].

These copper selenides also exhibit tunable localized surface plasmon resonances (LSPR) arising from high hole concentrations, with the strongest LSPR in Cu_2‐x_Se (0<x≤1) non‐stoichiometric compositions [20]. LSPRs for these materials are observed in the near‐infrared (NIR) region similar to copper sulphides [21], enabling their applications in various fields such as fiber optical communication in the NIR transparency window or as bio‐compatible materials. Similar to metal nanoparticles, the observed LSPRs of degenerately doped semiconductor NCs (for instance, Cu_x_Se) can be tuned via particle size [22], shape [23], and surface chemistry as well as by modulating the free carrier density and distribution within the NPs [24, 25]. Since the plasmonic characteristics of these nanomaterials are dependent on their size and shape, which in turn are influenced by the experimental procedure used, the systematic optimization of synthetic conditions is essential to obtain the desirable plasmonic properties.

The compositions and crystal structures of copper selenide NCs are crucial determinants of their properties. Various chemical approaches have been explored to synthesize 2D Cu_x_Se NCs. Notably, many reported routes rely on selenium precursors requiring elevated decomposition temperatures, such as trioctylphosphine‐Se (TOP‐Se) [26, 27], which favor thermodynamically stable phases [28]. However, in comparison with Cu_2_Se and Cu_2‐x_Se, relatively few studies have focused on 2D CuSe, despite its distinct crystal structure and highest concentration of quasi‐free holes among copper selenides. This distinction is important because Cu_2‐x_Se has a non‐layered structure with vacancy‐driven plasmonics, whereas CuSe possesses a quasi‐*layered* klockmannite structure that provides intrinsic hole density, strong dielectric anisotropy, and the potential for hyperbolic optical behavior not accessible in Cu_2‐x_Se. Several solution syntheses have been reported for quasi‐2D CuSe. Among them, OLA‐only syntheses are particularly notable [29, 30], as oleylamine can act as both solvent and reductant, however, these routes frequently yield mixed Cu_2‐x_Se/CuSe phases or require additional Cu^+^ injection to preserve nanosheet morphology, limiting phase purity and shape control [31]. Other strategies include ionic‐liquid Se precursors [32], microwave‐assisted syntheses producing broad lateral distributions [33, 34], and PVP‐assisted hot‐injection methods [4].

The synthesis of single‐phase CuSe is inherently challenging due to its complicated klockmannite structure and the intrinsic variable valence states of Cu and Se, compared to the Cu_2‐x_Se solution synthesis [35]. The klockmannite structure features alternating covalently bonded CuSe_3_‐Cu_3_Se‐CuSe_3_ layers with hypothesized Se–Se van der Waals layers along the z‐axis [31] or covalent bonds between chalcogens in 4e sites [36]. Thus, the bond valence of Cu with Se at all lattice sites remains debated. This structural complexity makes CuSe substantially more difficult to stabilize than Cu_2‐x_Se, which forms readily under similar conditions. To the best of our knowledge, a synthesis approach that efficiently produces CuSe NCs with uniform shape or size control is still lacking. Thus, an eco‐friendly, facile and effective solution method for synthesizing high‐quality klockmannite CuSe nanostructures is needed.

Herein, we report a simple, phosphine‐ and thiol‐free, effective hot‐injection synthesis of colloidal 2D CuSe nanocrystals. This method uses non‐coordinating 1‐octadecene (ODE) as the solvent and oleylamine as both the reductant and ligand, thereby completely avoiding the use of toxic, pyrophoric, and expensive alkylphosphines, as well as dodecanethiol. Thiol ligands can reduce Se, introduce sulphur impurities or promote Cu–S side reactions, whereas thiol‐free conditions avoid such competing pathways and enable cleaner growth of pure klockmannite CuSe [37]. The resulting nanocrystals form micron‐sized nanosheets (NSs) in a wide lateral size range up to 4 µm, and uniform triangular nanoplatelets (NPLs) in the size range of 12–25 nm. We also discuss the steady‐state and transient optical properties of our NCs, focusing on the influence of shape on plasmon resonances and absorption spectra, supported by advanced complex‐scaled discrete dipole approximation (CSDDA) simulations.

## 2 Materials and Methods

### 2.1 Chemicals

Unless stated otherwise all chemicals were ordered from Sigma‐Aldrich and used without any further purification. Copper (I) iodide (CuI, 99.5%), selenium powder (Se, ‐100 mesh, 99.99%), oleylamine (OLA, 70%), octadecene (ODE, 90%), toluene (99.5%), hexane (VWR, 95%), isopropanol (VWR, 99.7%) and acetone (Th. Geyer, 99%).

### 2.2 Synthesis of CuSe NCs

CuSe NCs were synthesized using a hot injection method under an argon atmosphere using a Schlenk‐line setup. Initially, 2 mmol to 4 mmol Se was added to a mixture of OLA and ODE with a 1:3 ratio (8 – 16 mL) in a three‐neck flask, and that mixture was heated at 200°C under an argon atmosphere till the Se was completely dissolved. Afterwards, the temperature was stabilized at 70°C and the solution was degassed by applying vacuum for 1 h. Then the flask was filled with argon and gradually heated up to the required synthesis temperature. Further, 3 mL of a previously degassed CuI/OLA solution (0.05 to 0.2 mmol of CuI) was hot injected at 200–220°C. Aliquots were taken during the reaction at different time scales to study the formation mechanism and growth of the NCs. The reaction mixture was refluxed between 5–10 min and then cooled down to room temperature by quenching it with a cold‐water bath. For purification, the crude CuSe NCs were precipitated by adding an acetone/isopropanol mixture and then extracted by centrifugation. The precipitated product was resuspended in hexane and the washing procedure was repeated twice. The final purified NCs were dispersed in hexane for further characterization use.

### 2.3 Characterization of the CuSe NCs

X‐ray diffraction (XRD) measurements were performed on a Malvern‐Panalytical Aeris System with an X‐ray wavelength of 0.154 nm from the Cu‐Kα1 line. The samples were prepared by drop‐casting the suspended NCs on a low background silicon wafer substrate (<911> or <711> cut). TEM samples were prepared by diluting the suspension with hexane followed by drop‐casting it on a copper grid with a carbon film. Standard TEM images and selected area electron diffraction (SAED) patterns were acquired on a Thermofisher Talos‐L120C microscope with a thermal emitter operated at an acceleration voltage of 120 kV. High‐resolution STEM images were acquired on a Jeol ARM20CF NeoARM microscope at 200 kV acceleration voltage. UV/Vis/NIR absorption spectra were obtained using a quartz cuvette with a Lambda 1050+ spectrophotometer from PerkinElmer equipped with a 150 mm‐large integration sphere. Transient absorption (TA) spectra were recorded with ≈100 fs resolution using a pump‐probe setup with a white light continuum for probing and a noncollinear optical‐parametric amplifier (NOPA) as excitation source (460 nm, ≈70 fs pulses) [38]. Both were pumped by a Ti:sapphire laser system (CPA 2001, Clark MRX) at 775 nm and a repetition rate of 1 kHz. The probe was dispersed by a prism and detected via a photodiode array. To minimize scattering effects, the pump and probe pulses were cross‐polarized to filter out the scattered pump light with a linear polarizer in front of the detector. Samples in hexane were drop‐cast onto quartz substrates resulting in optical densities of ≈ 0.15–0.25 at 460 nm. Raman spectra were obtained using a self‐built micro‐Raman spectroscopy setup on samples drop‐cast onto silicon wafer pieces with a top SiO_2_ layer. For the Raman measurements, a linearly polarized, diode‐pumped solid‐state laser with a wavelength of 532 nm was focused onto the samples using a 50x microscope objective to a spot size of about 1 µm. The backscattered light was collected by the same objective. The elastically scattered light was filtered out using a set of three reflective Bragg grating notch filters (Optigrate), giving access to Stokes‐ and Anti‐Stokes Raman signals. The inelastically scattered light was further analyzed using a linear polarizer to yield cross‐polarized detection and detected using a grating spectrometer and a Peltier‐cooled charge‐coupled‐device camera.

### 2.4 Band Structure and CSDDA++ Calculations

First‐principles computations were performed using the Questaal suite [39] for the band structure and optical response of CuSe. A modified complex‐scaled discrete dipole approximation [40] (CSDDA++) was applied to simulate field excitations within CuSe NCs, using the obtained dielectric functions. Our version enhances conventional DDA [41] by optimizing the iterative scheme in the complex plane, achieving faster convergence. Numerical simulations were conducted on the Zarquon Cluster (224 CPU threads) with cubic dipoles of 1.5 Å. For a 20 nm NC, over one million dipoles were used, covering a volume of 3456 nm^3^. Simulation times ranged from a few hours for the dielectric domain to 26–30 h for the hyperbolic domain. The resulting intensity‐normalized absorption spectra are presented in the Supporting Information for various shapes and field incidences, following Ref. [42].

## 3 Results and Discussion

### 3.1 Formation and Morphology of the Nanocrystals

The quasi‐2D CuSe nanostructures were synthesized using the hot‐injection approach followed by size‐selective precipitation. A schematic representation of the synthesis process is shown in Figure S1, and a detailed description of the synthesis technique is provided in the Experimental section. CuI was used as the Cu^+^ precursor to stabilize copper via soft Lewis acid–base coordination with iodide, thereby moderating its reactivity and favoring stoichiometric Cu_1_Se [43, 44]. In a typical synthesis, CuI in OLA (0.2 mmol) was injected into a Se solution (4 mmol) in ODE and OLA at 200°C without additional ligands. The combination of OLA and a selenium‐rich environment is the key factor directing the reaction toward the klockmannite CuSe phase and suppressing formation of non‐stoichiometric Cu_2‐x_Se. This ligand–anion environment moderates Cu^+^ reactivity and stabilizes the 2D phase.

**FIGURE 1:**
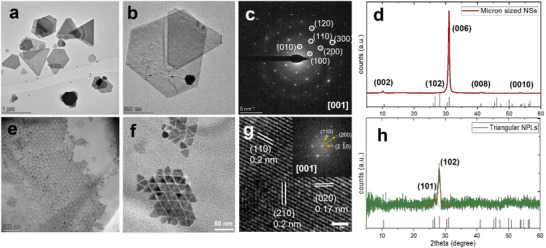
Structural characterization of the klockmannite CuSe nanostructures. (a,b)TEM images of micron sized NSs (c) SAED of single NS along the [001] zone axis (d) respective powder XRD of NSs. (e,f) TEM images of triangular NPLs (g) HRTEM of single NPL with FFT along the [001] axis (h)respective powder XRD of NPLS with fitted profile for (101) and (102) reflections. XRD reference PDF card No. 34–0171 of klockmannite CuSe phase was used.

The precipitate consisted of nanosheets (NSs) with a nonuniform size distribution, with lateral sizes of 0.2–4 µm and thicknesses of 5–15 nm as presented in Figure 1a,b. High‐resolution TEM (HRTEM) analysis and SAED revealed that these NSs have a hexagonal klockmannite crystal phase with *a = b* = 3.94 Å and *c* = 17.25 Å (Figure 1c,d). The SAED pattern is indexed along the [001] zone axis, confirming the single‐crystalline nature of the NSs. XRD further verified phase purity, with the diffraction peaks at 2θ = 10.1°, 27.8°, 31.0°, 41.9°, and 53.1° corresponding to the (002), (102), (006), (008), and (0010) planes of klockmannite CuSe. The reduced number of peaks and the strong (006) reflection arise from texture effects associated with preferential [001] orientation on the Si substrate, consistent with SAED observations and the simulated XRD pattern of a [001]‐oriented klockmannite crystal (Figure S2).

When gradually decreasing the Cu concentration in the synthesis, hexagonal NPLs (Cu:Se = 0.1:4 mmol) and later faceted triangular NPLs (Cu:Se = 0.05:4 mmol) with an average altitude size of 16 nm and thickness of ≤5 nm are formed together with micron‐sized NSs. Their 2D morphology is evident from homogeneous TEM contrast, HRTEM fringes, lateral overlap and stacking behaviour. These NPLs were separated using size‐selective precipitation and filtration procedure (Figure S1). After quenching the reaction, the crude product was centrifuged to collect the supernatant (containing NPLs and some small NSs) and the supernatant was purified via repeated low‐RPM (1500) centrifugation. Additional separation was achieved using 200 nm PTFE filters, which improved the separation and purity of the small NPLs. Across repeated syntheses, triangular NPLs were obtained with high reproducibility, comprising ∼50%–60% of the isolated nanocrystal fraction. Their size‐distribution histograms (Figure S1b) confirm relatively narrow dispersity (standard deviation of 12%) and support uniformity.

Hexagonal NPLs crystallize in the berzelianite phase with some contribution from large klockmannite NSs (Figure S3‐1), whereas triangular NPLs exhibit a pure klockmannite structure, as confirmed by XRD (Figure 1h) and HRTEM (Figure 1g). In triangular NPLs, HRTEM lattice fringes correspond to d‐spacings of ∼0.2 nm for the (110) planes and 0.17 nm for the (020) planes, indexed along the [001] zone axis. Formation of triangular klockmannite structures is highly sensitive to precursor mixing and the local redox environment, often leading to mixed or hexa‐triangular morphologies in the literature [45, 46]. In our case, the triangular shape originates from the anion‐rich environment created by excess selenium, which ensures Se‐terminated early nuclei, suppresses Cu‐rich intermediates and accelerates nucleation in the kinetic regime [47]. High local concentrations of reactive Se‐OLA species favour lateral 2D growth, enabling thin triangular plates with large surface‐to‐volume ratios. During growth, OLA acts as both reductant and ligand, passivating Cu cations due to the nucleophilic nature of the amine group. DFT studies for copper sulfide [48] show higher amine adsorption energies on Cu‐rich (100) facets than on (110), supporting by analogy the formation of triangular and hexagonal platelets with (100)‐like edge facets in CuSe.

In addition to the specific choice of surfactants, reaction temperature plays a critical role in controlling the phase of CuSe NCs. For instance, syntheses performed below 200°C yielded berzelianite phase (Cu_2‐x_Se) NCs with cubic morphology and uniform size distribution [49] (Figure S3‐2), while syntheses conducted over 230°C produced a berzelianite phase (Cu_1.8_Se) mixture with micron‐sized klockmannite NSs (Figure S3‐3). The formation of various Cu_2‐x_Se phases merits further investigation, as this system can crystallize into multiple closely related structures with small differences in formation energy [33], each capable of accommodating a broad range of copper stoichiometries [50]. In our study, we find that only a narrow range of experimental conditions promotes the formation of the “metastable” klockmannite phase of copper selenide.

#### 3.1.1 Metallicity of CuSe NSs

CuSe is known as a semiconductor with high p‐type conductivity, demonstrated in both large‐scale films [51, 52] and, more recently, in films of nanoparticles or nanosheets [6, 19, 31, 33, 53]. The origin of its metallic character can be attributed to the mixed Cu/Se valence states (+1, +2 / 0, ‐2), arising from strong Cu *d* – Se *p* hybridization, and to the presence of delocalized holes in the valence band, analogous to covellite copper monosulfide.

While films of CuSe have been extensively studied, individual colloidal NSs had not previously been contacted and electrically characterized. To investigate this, we contacted several individual NSs using e‐beam lithography and observed nearly linear I–V curves with metallic behaviour, i.e. a positive temperature coefficient of resistivity (using cryo‐probe station), which is characteristic for a metallic behavior. The values of the specific conductivity were 645.2 (Ω∙cm)^−1^ at 293 K and 1266.5 (Ω∙cm)^−1^ at 5 K (Figure S4), consistent with previously reported values for continuous films and nanoparticles. Our measured room temperature conductivity is approximately half of that reported for thin films in Refs. [51, 52]. This discrepancy can be attributed primarily to the residual L‐type surface ligand oleylamine, whose nucleophilic and electron‐donating tether group [54] can attract and partially block quasi‐free holes at the nanocrystal surface. In addition, contact resistance induced by residual ligands, enhanced charge scattering at the surfaces of thin NSs, and a reduced channel width likely contribute to the reduction in conductivity. Nevertheless, the measured values remain competitive for various applications and are comparable to those of highly conductive polymers. More detailed insight about the electrical properties of CuSe NSs will be given elsewhere.

### 3.2 Optical Steady‐State Properties of CuSe NCs in the Visible and NIR Regions

**FIGURE 2:**
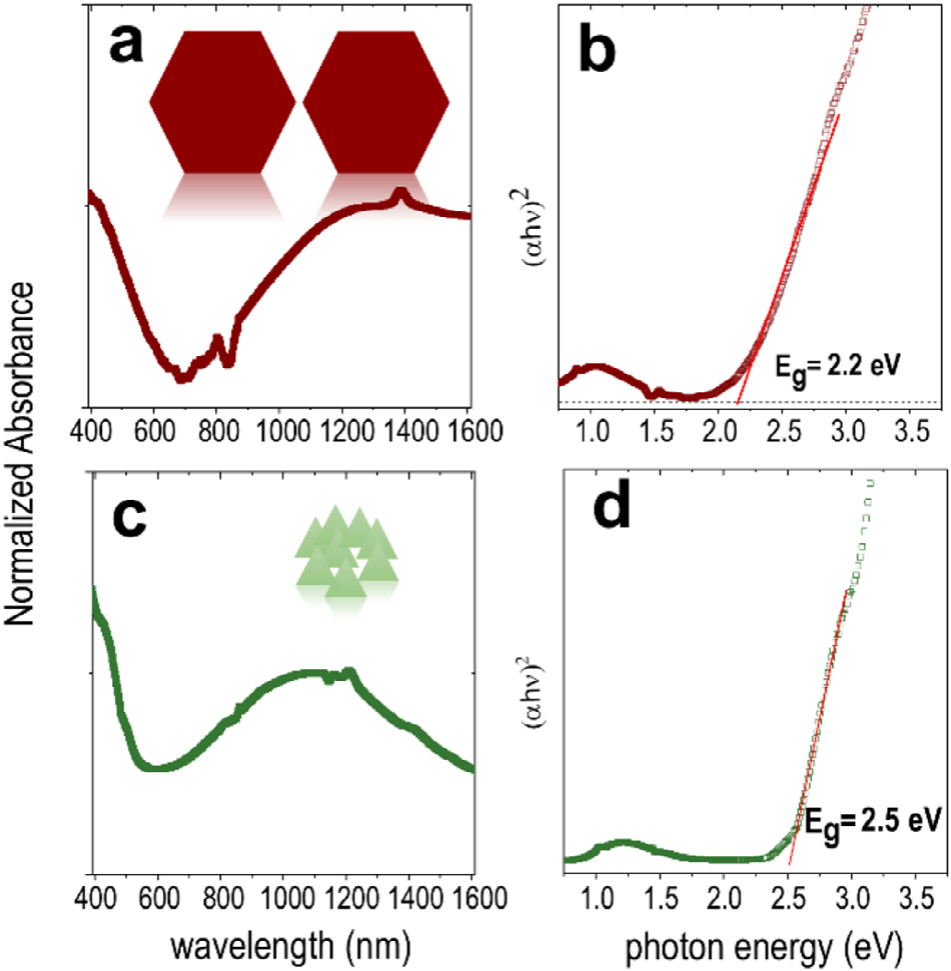
(a, c) Absorption spectra of nanosheets in red and triangular nanoplatelets in green. (b, d) Tauc plots of the respective spectra considering CuSe as direct band‐gap semiconductor.

Along with their high electrical conductivity and controlled morphology, the optical properties of CuSe deserve detailed examination. Figure 2 shows typical optical absorption spectra of CuSe NCs suspended in hexane and measured in a quartz cuvette. A broad absorption feature appears between 400–600 nm and extends as a tail into the ultraviolet. The optical band gaps of ∼2.5 eV for NPLs and 2.2 eV for NSs were estimated using Tauc linearisation by plotting of (*αhν*)^2^ versus photon energy (*hν)* (shown in Figure 2b,d, where *α* is the absorption coefficient) assuming a direct band gap [55]. The blue‐shifted band gap of the NPLs relative to bulk CuSe (2.2 eV) [8], likely reflects a combination of the Moss–Burstein and quantum confinement effects [20].

Many cation‐deficient copper chalcogenides are degenerately doped semiconductors, tolerating a large number of ion vacancies and thus high charge carrier concentrations [56, 57]. As discussed earlier, klockmannite CuSe supports a high density of quasi‐free holes due to its specific crystal structure, similar to CuS as established through theoretical and particularly DFT studies [58, 59, 60, 61]. This allows CuSe NCs to sustain high intensity LSPRs, particularly in the NIR region [62] while shape and size tunability provide extensive control over these resonances. Freshly prepared CuSe NSs exhibit a broad, nonsymmetric plasmon band peaking at around 1400 nm, whereas smaller triangular NPLs (average size ∼12–25 nm) display a narrower peak at ∼1100 nm. Similar features are found for hexagonal NPLs in the cubic (isotropic) berzelianite phase (Figure S5), which also exhibit strong localized surface plasmon bands in the NIR due to copper vacancies. The redshift of the bands in NSs with respect to NPLs is attributed to the larger lateral size resulting from slower and more balanced growth. Sharp features in the spectrum in the NIR originate from the measurement (solvent) artefacts.

Since both triangular NPLs and large NSs crystalliz [e in the klockmannite phase, it is important to analyse their plasmonic properties in the NIR while accounting for anisotropy. Simulating LSPRs in irregular or complex geometries requires numerical methods, with the dielectric permittivity dispersion as a key input. This dispersion can be approximated within the Drude–Sommerfeld theory [48, 63]. An essential drawback of this approach for CuSe is the necessity of several fitting parameters (plasma frequency, damping constant, bound electrons polarizability parameter *ε_∞_*), the absence of fundamental absorption and – more importantly – the absence of anisotropy. A more rigorous alternative is to obtain the band structure and construct the full permittivity tensor.

### 3.3 DFT Calculations

The band structure of CuSe was calculated using the quasiparticle self‐consistent GW (QSGW) method, and the permittivity along two crystallographic directions (in‐plane and out‐of‐plane) was derived using the random phase approximation (RPA) following the approach of Zayats et al. [42]. These optical parameters were then used in CSDDA simulations to model the near‐field distributions around CuSe nanotriangles.

**FIGURE 3:**
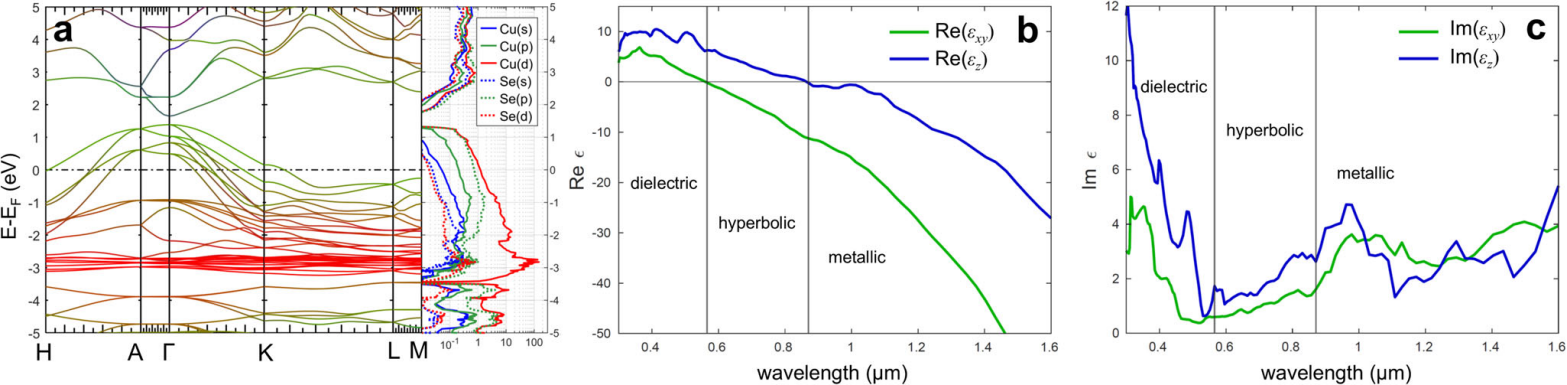
Electronic band structure and optical survey of CuSe using the QSGW‐RPA approximation: (a) band structure with color coding: blue—*s* orbitals of Cu and Se, green—*p* orbitals and red—*d* orbitals, inset – projected density of states in CuSe, the Fermi level is set to 0 eV, (b) real part and (c) imaginary part of the dielectric function along xy (in‐plane) and z direction (out‐of‐plane, i.e. [001] direction).

Figure 3a shows the CuSe band structure obtained from the QSGW+RPA method, with the Fermi level set to 0 eV (actual value: 0.479 eV). The Fermi level lies within the valence band, confirming that CuSe is a p‐type semiconductor with a direct band gap of 0.257 eV at the Γ‐point. The valence band originates from hybridisation between Cu d‐orbitals and Se p‐orbitals, while the conduction band derives from their respective s‐states. Hole dispersion is minimal along the Γ–A ([001]) direction but significantly larger along Γ–K (in‐plane), highlighting the strong electronic anisotropy of CuSe. Likewise, the conduction band shows its highest dispersion along Γ–K. The effective masses of the holes, extracted from the band dispersion, are 0.86·me in the xy‐plane and 1.05·me along z, inclusion of spin–orbit coupling further increases this discrepancy.

The dielectric function *ε(ω)* of CuSe was calculated over a broad energy range from 0.8 up to 4 eV (Figure 3b,c). In Figure 3b, the real part of the dielectric function shows that CuSe exhibits a dielectric response in the Vis range below 550 nm where Re[ε(ω)] is positive and Im[ε] high (Figure 3c). In the NIR range above 900 nm, both out‐of‐plane and in‐plane components of Re[ε(ω)] are negative and the light propagation is forbidden, making the material metallic. In the spectral range from nearly 550 to 880 nm, a hyperbolic optical response exists, where the permittivity in the xy‐plane (Re[ε_*xx*_], Re[ε_yy_]) has an opposite sign to that along the z‐axis (Re[ε_*zz*_]). This hyperbolicity extends partially into the visible and the NIR regions. The existence of the hyperbolic range in the vicinity of the plasma frequency is expected for anisotropic materials since the effective masses are different for in‐ and out‐of‐plane directions. From classical Drude theory it is known that the plasma frequency is a parameter which defines the zero‐crossing point of the permittivity. After *ε* becomes negative, propagation of light in the medium is shielded by the plasma oscillation and the medium becomes strongly reflective. Being a function of the charge carrier concentration *N* and their effective mass m^*^ ($\omega_{p}={\left[\frac{eN}{\varepsilon_{0}m^{*}}\right]}^{\frac{1}{2}}$), the plasma frequency becomes a tensor for anisotropic media allowing for a hyperbolic domain. We note that the absolute position of the zero‐crossing points of the permittivity tensor based on DFT calculations is an approximation, however, the emergence of the hyperbolic domain is a principal result with a higher degree of plausibility.

**FIGURE 4:**
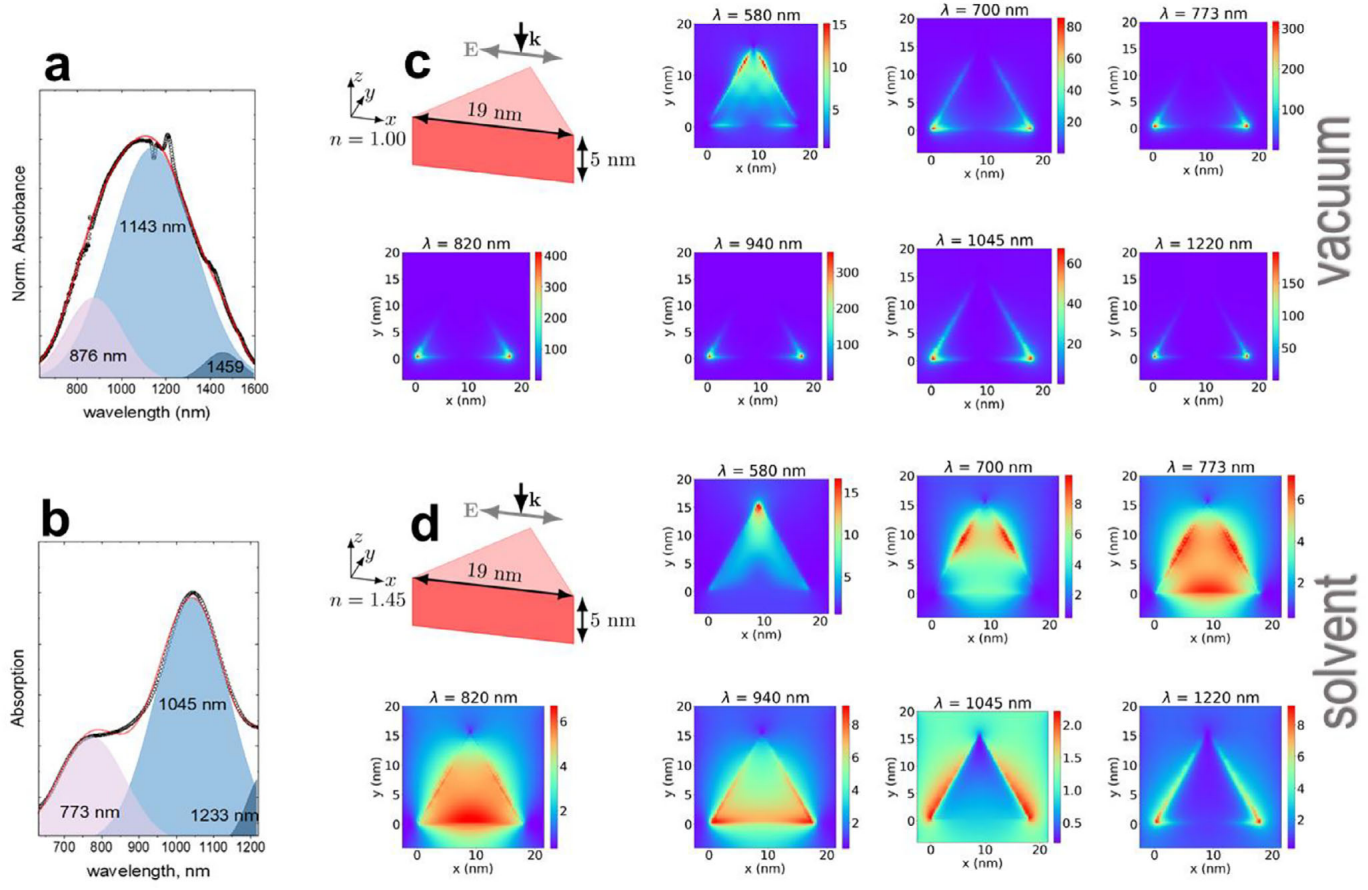
(a) Experimental absorbance of the triangular CuSe NPLs in solution (n≈1.45) and (b) simulated orientation‐averaged absorption cross‐section (nm^2^) of the triangular CuSe NPLs. (c, d) Predicted spatial electric field intensity distributions 0.1 nm above the surface of the NPL for different media around the NPL. (c) vacuum (no solvent), (d) with a solvent (n = 1.45).

With the obtained dielectric function, the plasmonic responses to incident fields were further studied in detail for CuSe NCs. The intensity‐normalised absorption spectra calculated from CSDDA++ (see details in SI) for triangular NPLs are shown in Figure 4 and in Figure S6 for other possible shapes for comparison. The edge length of the NPLs in the simulations is 19 nm and the thickness is 5 nm.

During the simulations, we averaged the absorption spectra for changing polarization with respect to the plane of the nanoprism (idealised model geometry for triangular NPLs) surface in three directions (so‐called “orientational averaging”). First, we consider the collective spectrum of triangular NPLs experimentally measured in hexane (Figure 4a). For qualitative understanding, we fitted the absorbance curve with three Gaussian functions. A similar procedure has been performed for the calculated spectrum in Figure 4b. In the measured spectrum, the main contribution comes from the central peak at ∼1140 nm, attributed to the strongest LSPR mode which is dipolar. Side peaks are needed to compensate for the residual absorption and asymmetry characteristic of the triangular NPLs. The long‐wavelength component is likely coming from a size distribution shifted towards slightly larger particles, while the short‐wavelength contribution likely arises from quadrupolar resonance or out‐of‐plane modes. Considering the anisotropy of CuSe in that spectral range and the predicted hyperbolicity, the out‐of‐plane mode can be ruled out. In the calculated spectrum (Figure 4b), a dominant NIR LSPR appears at 1045 nm, accompanied by a higher‐energy shoulder near 773 nm. The sensitivity of this shoulder to nanocrystal orientation is illustrated in Figures S7–S9. The main peak in the measured spectrum (∼1143 nm) aligns closely with the simulated peak (∼1045 nm), red‐shifted by only ∼0.1 eV despite no fitting parameters. The high‐energy shoulder in the experiment is weaker and slightly more red‐shifted (∼0.2 eV) than in the simulations. Additional spectra (Figures S6 and S8) reveal that this short‐wavelength feature depends strongly on corner geometry and disappears for disk‐like platelets. This suggests that the vertices of the experimental triangular NPLs are not perfectly sharp but slightly rounded. Indeed, the corner curvature radius is estimated to be 2.8–4 nm for the triangular NPLs with an average edge size (Figure S10). Among further factors that can broaden, shift, or quench resonances chemical interface damping can be mentioned due to the presence of a monolayer of oleylamine on the surface [64]. Finally, collective ensemble effects such as size distribution, NC stacking, orientational disorder, light scattering, and additional LSPR damping from surface traps may influence the measured spectra and are not yet included in our simulations.

In Figure 4c,d, intensity profiles above the surface of the triangular NPL at different wavelengths (in cgs units, i.e. statV/cm^2^) are shown for different surroundings – vacuum and solvent (corresponding to the experiment *n* = 1.45) and solvent+substrate (SiO_2_) in Figure S11 for comparison. For the NC in vacuum, the resulting plasmonic excitations are tightly confined to the corners of the NC along the whole spectrum (Figure 4c). These spatial field distributions are similar to what one would expect in an isotropic medium. The resulting field enhancement is high. When the solvent is introduced (Figure 4d), wavelengths beyond 900 nm—corresponding to the fully metallic regime—produce dipolar‐like field distributions localised near the corners (at λ = 1045 and 1220 nm), characteristic of the LSPRs commonly observed in metals [65] and even for copper sulfide [42, 48]. In the hyperbolic domain, however, a significant deviation from the spatial intensity distribution of (c) is noted. Specifically, in the hyperbolic domain from 890 nm to 550 nm, the plasmonic excitation spreads beyond the vertices. Near the epsilon‐near‐zero wavelength (∼820 nm), an “envelope” excitation mode forms, covering nearly the entire surface of the NC, with notable enhancement along the edges. This hybrid mode combines quadrupolar features with an envelope‐type excitation. The predicted effect of the solvent + an infinite‐plate substrate can be found in Figure S9b. The addition of the infinite plate has a subtle but distinct effect on the plasmonic excitation at 820 nm from configuration (d) in Figure 4, suppressing the edge excitations. The plasmonic mode is now tightly confined within the edges of the NC in the hyperbolic domain. Simulations of absorption spectra for other shapes such as truncated triangles, hexagons and disks are also shown in the SI for comparison. We assume that in the hyperbolic spectral domain, a large range of evanescent modes on the surface of the nanocrystals in a solvent is allowed, and the field distribution gradually progresses towards the “envelope” mode on the entire surface of the NC as a result of a combination of propagating and evanescent fields. To disentangle the roles of shape and intrinsic crystal anisotropy, we simulated an isotropic “model” CuSe nanoprism (Figure S12). These simulations reveal that shape anisotropy determines the spectral shifts, hotspot locations, and asymmetric spectral broadening, whereas the intrinsic material anisotropy is solely responsible for the envelope‐type modes, which appear only in the anisotropic nanocrystal.

### 3.4 Ultrafast Dynamics of Charge Carriers in the Excited state

Along with their unique stationary optical properties, the ultrafast behavior of copper chalcogenides has also gained increasing attention in recent years. Relaxation and recombination dynamics are particularly crucial for these nanocrystals in energy‐transfer related applications. According to available studies on CuS [17, 66, 67, 68, 69, 70, 71], these materials differ from metals and semiconductors in their ultrafast relaxation dynamics due to their hybrid properties combining the fundamental band gap, specific concentration of quasi‐free charge carriers (very high for semiconductors and low in comparison to metals), surface phenomena and geometrical constraints imposed by synthesis conditions. In this regard, possible applications include but are not limited to hot‐carrier extraction, ultrafast switching and photodetection.

**FIGURE 5:**
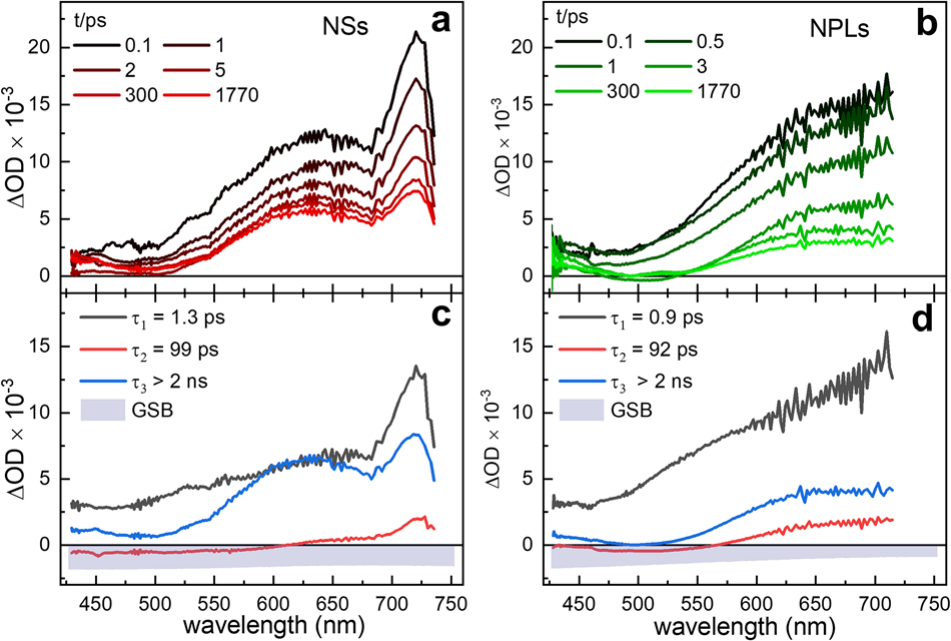
(a, b) TA spectra of NSs and NPLs at different times t after optical excitation at 460 nm. (c, d) DASs for NSs and NPLs obtained by the GLA and labelled by the respective time constants. The dynamics is well described by a multi exponential decay with the time constants τ_1_ = 1.3 ps, τ_2_ = 99 ps and τ_3_ > 2 ns for NSs and the time constants τ_1_ = 0.9 ps, τ_2_ = 92 ps and τ_3_ > 2 ns for NPLs.

To explore these properties further, we performed ultrafast pump‐probe transient absorption (TA) measurements on anisotropic klockmannite CuSe NCs, exciting the samples in the fundamental absorption region at 460 nm and tracking the induced absorption changes in the visible spectral range. As shown in Figure 5a,b, the TA spectra are dominated by a broad excited‐state absorption (ESA) that overlaps with the ground‐state bleach (GSB). The spectral shape and position of the GSB, indicated in grey in Figure 5c,d, are obtained from the respective steady‐state spectra of the NCs presented in previous sections.

To analyse the TA data Δ*OD*(λ, *t*), we performed a global lifetime analysis (GLA), fitting the transient spectra with a sum of exponential decay components using the function:

$$\Delta OD\;\left(\lambda ,t\right)=\sum_{i}A_{i}\left(\lambda \right)\cdot \exp\left(-\frac{t}{\tau_{i}}\right)$$

The *i*‐th decay component is characterized by its time constant τ_*i*_ and the decay associated amplitude spectrum (DAS) *A_i_* (λ). The obtained time constants are summarized in Table 1, and the corresponding DASs for NSs and NPLs are shown in Figure 5c,d, respectively. For both samples, the observed dynamics consists of three components: a fast, a medium‐fast, and a slow one. We interpret these results assuming that CuSe behaves similarly to the covellite CuS, which exhibits comparable mixed‐valence character, plasmonic response, and ultrafast relaxation characteristics.

**TABLE 1:** Time constants obtained by GLA of the TA data as well as frequencies and damping constants of the observed oscillations.

	*τ_1_* [ps]	*τ_2_* [ps]	*τ_3_* [ns]	*f* [THz]	*τ_d_* [ps]
NSs	1.3	99	>2	7.5	0.5
NPLs	0.9	92	>2	7.8	0.7

The shortest time constant *τ_1_* is attributed to the hole trapping process [17, 70, 72], occurring slightly faster in NPLs (*τ_1_* = 0.9 ps) compared to NSs (*τ_1_* = 1.3 ps). The two longer time constants *τ_2_* and *τ_3_* reflect a combination of photocarrier recombination and the relaxation of trapped holes [17, 70, 71, 72]. In both cases, the component with *τ_2_* (99 ps for NSs and 92 ps for NPLs) contributes only weakly and reflects a slight narrowing of the ESA signal in the red spectral region and a minor increase in the blue spectral region. The final decay occurs on the nanosecond timescale and is described by the time component *τ_3_*. Due to the experimental setup, which is limited to a maximum delay of 1.8 ns, an exact value for *τ_3_* cannot be determined. However, the data suggest that the decay in NSs is slightly slower than in NPLs. The tendency toward shorter time constants can be explained by the fact that smaller NPLs generally have higher defect densities and surface‐to‐volume ratios than NSs. Defects can serve as recombination centers, which accelerate the relaxation dynamics [73, 74].

**FIGURE 6:**
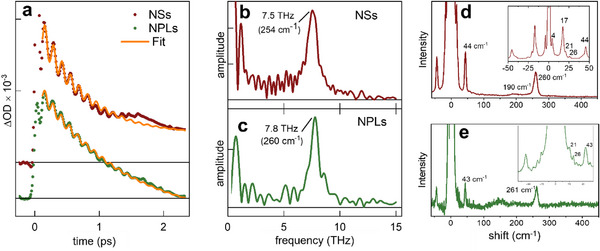
Oscillatory TA features and corresponding Raman spectra, (a) TA time traces averaged from 480 nm – 500 nm from NSs (red) and NPLs (green dots) with corresponding fits (orange curve). (b and c) FFT spectra of the residuals resulting from subtracting the exponential contributions from the averaged time traces for the samples NSs and NPLs. (d and e) Raman spectra of the CuSe NCs on a Si/SiO_x_ substrate in full and reduced spectral range (zoomed) for NSs (red curve) and NPLs (green curve).

Particularly interesting are the oscillatory features observed in the TA time traces during the first two picoseconds (Figure 6a). The ultrafast excitation of hot holes and electrons is accompanied by the generation of coherent phonons [69, 75]. To analyze these oscillations in more detail, the time traces in the wavelength range of 480–500 nm were averaged, and the first 2 ps were isolated. The underlying exponential decay was subtracted to extract the oscillatory components, and a fast Fourier transformation (FFT) was applied to the resulting residuals. The results, presented in Figure 6b,c for NSs and NPLs, reveal a pronounced mode at 7.6 THz for NSs and at 7.8 THz for NPLs. These modes can be confirmed by the measured Raman spectra of the samples (Figure 6d,e), which show vibrational modes at about 260 cm^−1^ assigned to the Se–Se stretching A_1g_ mode [76]. The oscillatory features can also be reproduced using a model containing two exponential decay terms and a damped oscillatory component. The oscillation frequencies extracted from the fits are 7.8 and 7.5 THz (Table 1, see Supporting Information for details), in good agreement with the FFT analysis. Interestingly, the NPLs exhibit a slightly larger damping constant compared to the NSs.

The phase of the oscillation can provide insight into the mechanism of coherent phonon generation [77]. However, the phase values obtained from the fit are not sufficiently precise, as the time‐zero of our measurements is accurate only to ±30 fs, corresponding to approximately a quarter of an oscillation period. We attribute the formation of the coherent phonons to the displacive excitation of coherent phonons (DECP) mechanism [69], in which photoexcitation induces a sudden shift in the equilibrium geometry, leading to coherent lattice vibrations [77]. An alternative mechanism would be impulsive stimulated Raman scattering (ISRS). DECP produces coherent vibrations in the electronically excited state, whereas ISRS generates them in the ground state. Evidence supporting DECP arises from the fact that the oscillatory features in the TA data exhibit slightly lower frequencies than those obtained from Raman spectroscopy, which probes the ground state. Because bonds in the excited state are typically weaker, the corresponding vibrational frequencies are expected to be lower, consistent with our observations. We further averaged the FFT data across different spectral regions. One range spans the absorption minimum between the stationary UV absorption and the plasmon band, while the other covers the adjacent flank of the broad UV absorption. Our reasoning is that if the oscillations originated from ground‐state vibrations, the stationary absorption spectrum should shift periodically with the vibrational frequency. In that scenario, the FFT amplitude would be strong on the spectral flank and weak in the absorption minimum. However, this behavior is not observed, instead, the FFT amplitudes are comparable across all analyzed regions (Figure S13). This provides additional evidence that the observed wave packets are generated in the excited state.

Lastly, considering the Raman spectra, we note, besides the already discussed Se–Se stretch mode at 260 cm^−^^1^, a weak mode at 190 cm^−^^1^ that likely corresponds to a Cu–Se stretch vibration, similar to that reported for CuS [78]. Additional lower‐frequency features, clearly identifiable on both the Stokes and anti‐Stokes sides, include modes at ±44 cm^−1^, ±26 cm^−1^, ±21 cm^−1^, ±17 cm^−1^ for NSs, and ±43.5 cm^−1^, ±26 cm^−1^, ±21 cm^−1^ for NPLs. The ±4 cm^−1^ mode should be assigned to Brillouin scattering of the LA mode of the Si/SiO_x_ substrate [79]. Features at 17 cm^−1^ and 45 cm^−1^ have been reported by Ishii et al. [76] and mentioned by Peiris et al. [36] in a CuSe compression study (though without interpretation). However, the remaining modes likely represent new low‐frequency, unassigned Raman features and might correspond to rigid‐layer shear and breathing modes, revealing the distinct (supposedly soft) interlayer coupling [73] in the quasi‐layered structure of CuSe. The detection of coherent lattice oscillations together with the corresponding Raman signatures provides strong evidence for the klockmannite phase in both the CuSe NSs and triangular NPLs, and confirms their pronounced crystal anisotropy. While the excitation of coherent phonons in CuSe is only indirectly related to its anisotropy (the Se–Se A_1_g stretch mode being aligned with the six‐fold [001] axis of klockmannite), the emergence of the hyperbolic domain is a direct consequence of the intrinsic structural anisotropy. Thus, together with their plasmonic response in the NIR, anisotropic CuSe nanocrystals represent a promising material platform for advanced optoelectronic and photonic applications.

## 4 Conclusions

In this work, we presented a thiol‐free colloidal synthesis of CuSe nanocrystals from nanoplatelets to micron‐sized large nanosheets and demonstrated a certain shape control including hexagonal and triangular nanoplatelets. We investigated various properties of these structures, including metallic electrical conductivity at different temperatures, as well as their optical and structural characteristics. Using GW DFT and CSDDA simulations, we derived absorption spectra that align well with experimental data, showcasing the material's broad tunability – fundamental absorption in the visible (Vis) range, plasmonic activity in the near‐infrared (NIR), and hyperbolicity across the Vis‐NIR spectrum. This hyperbolic behavior leads to unique field distributions, including enveloping surface modes, which result from the interaction between propagating and evanescent fields. This understanding opens up the possibility for engineering the optical response based on particle shape, orientation, and excitation wavelength. Additionally, we analyzed the ultrafast carrier relaxation and recombination dynamics in both nanosheets and nanoplatelets and observed a hot hole trapping process and the generation of coherent phonons, in agreement with Raman spectra. The Raman analysis also suggests a significant degree of anisotropy in CuSe, providing further insight into its unique properties and potential for advanced applications.

## Conflicts of Interest

The authors declare no conflicts of interest.

## Supporting information




**Supporting File**: smll72575‐sup‐0001‐SuppMat.pdf.

## Data Availability

The data that support the findings of this study are available from the corresponding author upon reasonable request.
