# Supplementary material for: Unlocking Klockmannite: Formation of Colloidal Quasi‐2D CuSe Nanocrystals and Photo‐Physical Properties Arising From Crystal Anisotropy
Source: Small. 2026 Jan 29;22(17):e12836. doi: 10.1002/smll.202512836 (PMC13003283; doi:10.1002/smll.202512836)
Supplement: Supplementary file 1 — Supporting File: smll72575‐sup‐0001‐SuppMat.pdf. [file SMLL-22-e12836-s001.pdf]

Supporting Information

Unlocking klockmannite: formation of colloidal quasi-2D CuSe nanocrystals and photo-physical properties arising from crystal anisotropy

Urvi Parekh[1], Nadiia Didukh[1], Samira Dabelstein[1], Ronja Piehler[1], Eugen Klein[1], Jivesh Kaushal[1], Tobias Korn[1], Stefan Lochbrunner[1,2], Christian Klinke[1,2], Stefan Scheel[1]*, Rostyslav Lesyuk[1,3]*

[1] Institute of Physics, University of Rostock, Albert-Einstein-Straße 23, 18059 Rostock, Germany
[2] Department "Life, Light & Matter", University of Rostock, Albert-Einstein-Straße 25, 18059 Rostock, Germany
[3] Pidstryhach Institute for applied problems of mechanics and mathematics of NAS of Ukraine, Naukowa str. 3b, 79060 Lviv, Ukraine

* corresponding authors: stefan.scheel@uni-rostock.de, rostyslav.lesyuk@uni-rostock.de

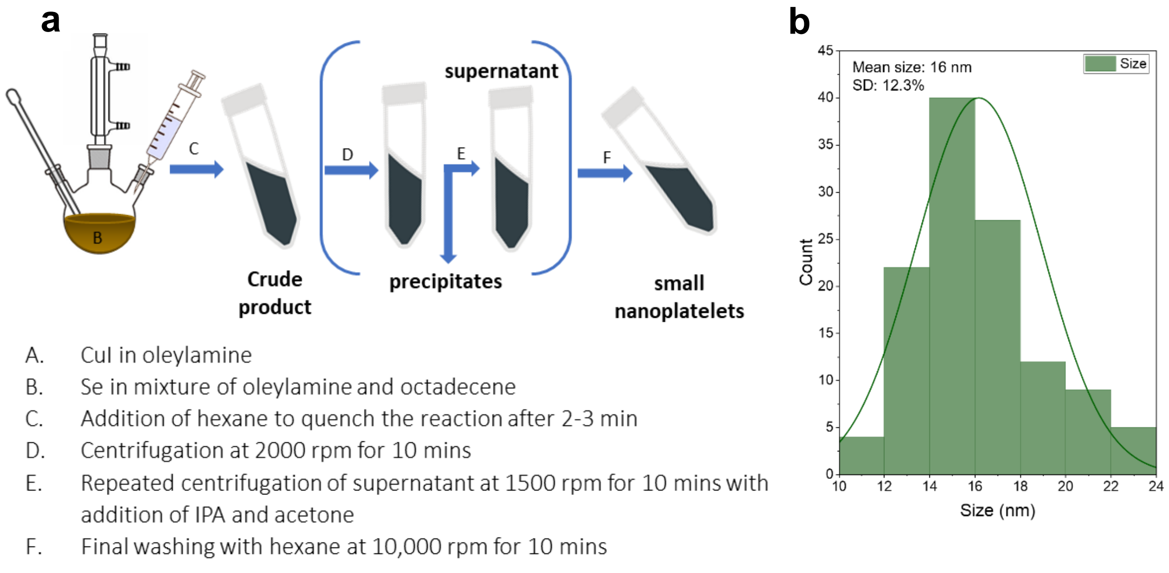

Fig. S1. (a) Synthesis procedure of CuSe nanosheets with size-selective precipitation of nanoplatelets and (b) histogram showing the size distribution of nanoplatelets (altitude of the triangles).

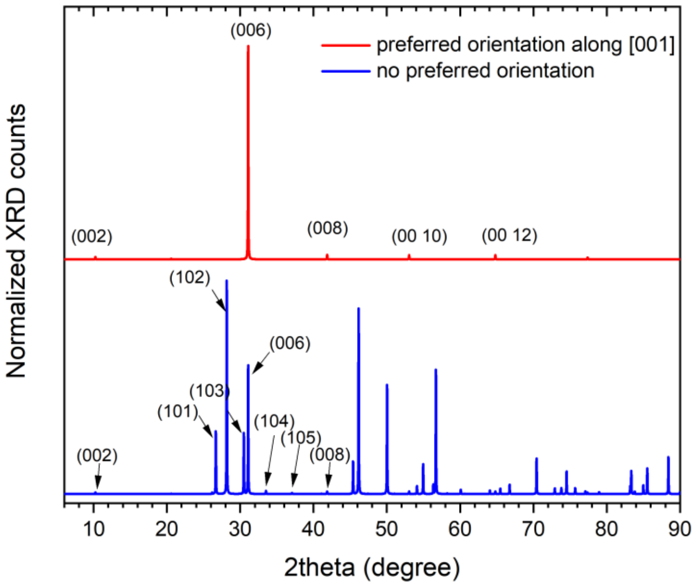

Fig. S2. Calculated XRD patterns of klockmannite CuSe crystal based on the crystallographic data of L.G. Berry [The crystal structure of covellite, CuS and klockmannite, CuSe American Mineralogist, 1954, 39, 504-509]. PowderCell 2.4 was used for the simulation[1].

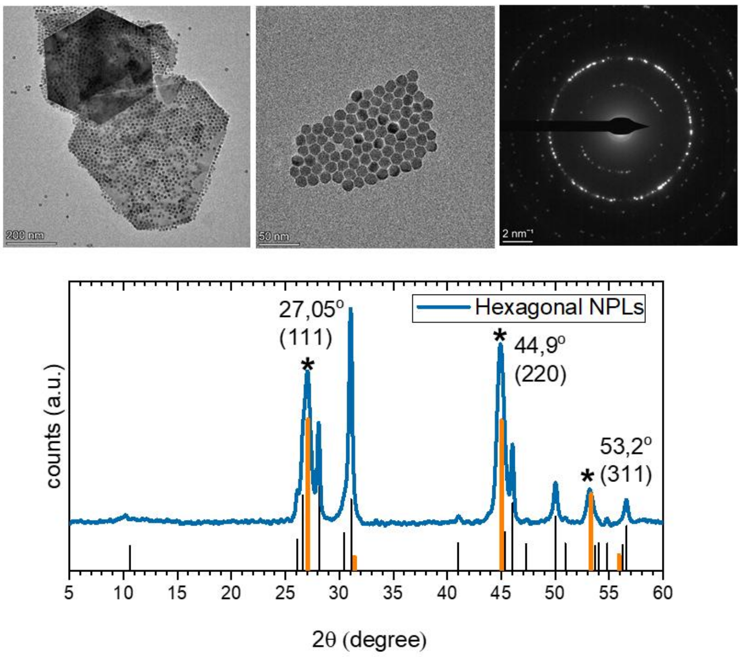

Fig. S3-1. Hexagonal NCs: TEM images, SAED and XRD pattern. Reflections marked with asterisk belong to the berzelianite phase (PDF card No. 01-088-2043).

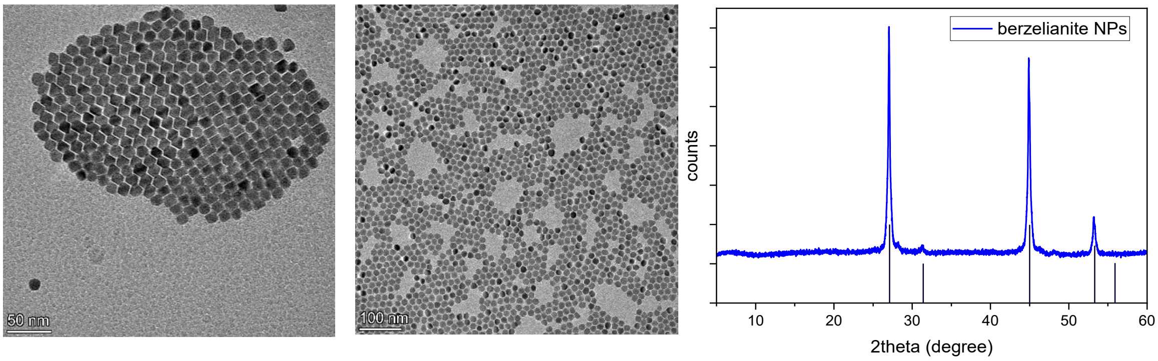

Fig. S3-2. Synthesis done under 200°C resulting in NPLs purely with berzelianite phase

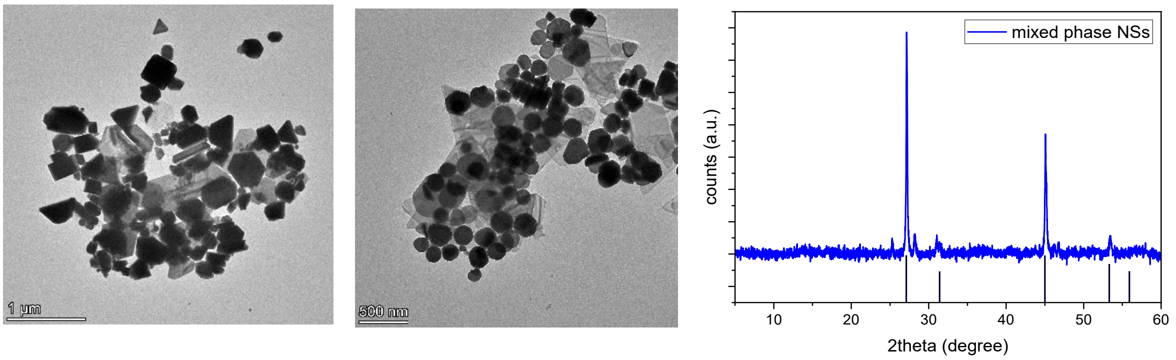

Fig. S3-3. Synthesis done over 220°C resulting in thick NSs of mixed phase predominantly containing berzelianite phase

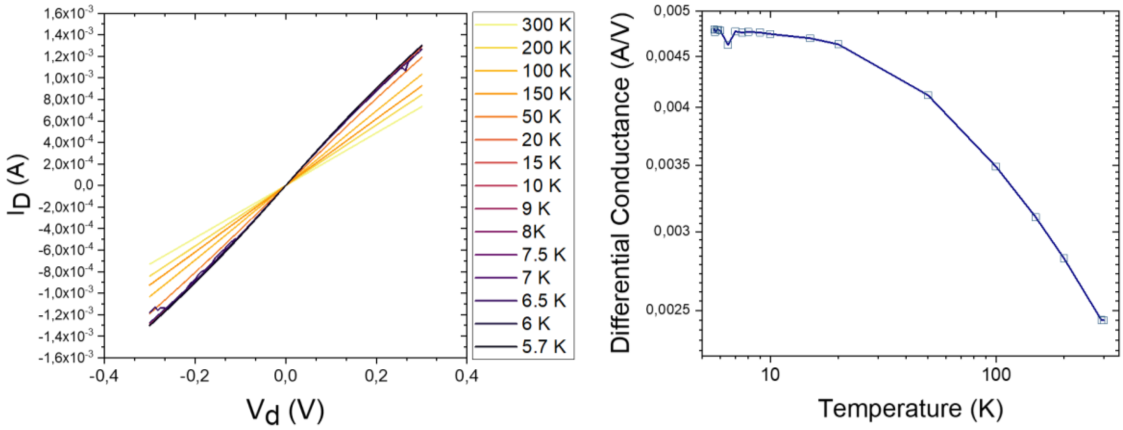

Fig. S4. Electrical transport through individual CuSe NSs

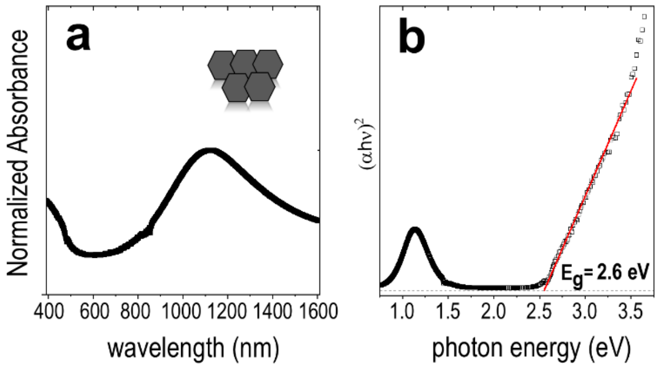

Fig. S5. Absorption spectra of hexagonal berzelianite NPLs with cubic crystal structure and respective Tauc plot. A strong plasmon band is present in the NIR.

QSGW simulations

To determine the optical properties of CuSe NCs in detail, quasi-particle self-consistent GW (QSGW) simulations and scattering calculations using the discrete-dipole approximation (DDA) were performed. To do so, we calculate the complex dielectric function *ε* over a wide spectral range. We employ a method used previously by Zayats et al. [2] to extract the complex *ε* of CuSe for two crystallographic directions (in-plane and out-of-plane) related to the strong anisotropy in this material.

First, the band-structure and optical response of bulk CuSe has been derived using the QSGW approach. Conventional density-functional theory (DFT) methods can deliver variety of structural, electronic, mechanical and thermodynamic properties [3–6] however they frequently lead to a band structure with the absence of a band gap. Along with time-dependent DFT based on solving a time-dependent Schrodinger equation with single-body electron density as a fundamental variable, the GW approximation (GWA)[7,8] presents another category of approximations that are based on many-body perturbation theory. It considers the exchange-correlation energy as a series of interactions involving multiple particles using one particle Green function (G) and screened Coulomb interaction (W) [9,10].

Quasi-single-particle GW (QSGW) gives a fairly accurate and qualitatively correct assessment of phenomena in most physical systems, in particular for CuSe, and together with random phase approximation (RPA) [2], it takes into account the plasmonic nature of the system.

Complex-scaled discrete-dipole approximation (CSDDA) Method

Here, we present the details of implementing the CSDDA algorithm. We first consider the standard recursive iteration at the heart of all DDA schemes. For the n_{th} dipole, the local field is iteratively calculated using the algorithm [11]:

$$\boldsymbol{E}_{loc,n}^{(i+1)} = g^{(i)} \left(\boldsymbol{E}_{inc,n} + \boldsymbol{E}_{scatsum,n}^{(i)}\right) + (1 - g^{(i)})\boldsymbol{E}_{loc,n}^{(i)} \quad (1)$$

where $\boldsymbol{E}_{loc,n}^{(i)}$ is the local field at the nth dipole, $\boldsymbol{E}_{inc,n}$ is the incident field at the nth dipole, $\boldsymbol{E}_{scatsum,n}^{(i)}$ is the scattered field at the nth dipole due to all other dipoles,

$$\boldsymbol{E}_{scatsum,n}^{(i)} = \sum_{\substack{m=1 \\ m\neq n}}^{N} \boldsymbol{G_{nm}} \cdot \boldsymbol{E}_{loc,m}^{(i)}. \quad (2)$$

In Eq. (1), $g^{(i)}$ is an optimization parameter at iteration i. With CSDDA, the goal is to find a value of $g^{(i)}$ at every iteration step i, so that the relative error $R^{(i)}$,

$$R^{(i)} = \sqrt{\frac{\sum_{n=1}^{N} |\boldsymbol{E}_{inc,n} + \boldsymbol{E}_{scatsum,n}^{(i)} - \boldsymbol{E}_{loc,n}^{(i+1)}|^2}{N}} \quad (3)$$

has the minimum possible value. This is done by choosing the value [12]: $g^{(i)} = -\frac{\sum_{n=1}^{N} \boldsymbol{X}_n^{(i)*} \cdot \boldsymbol{Y}_n^{(i)}}{\sum_{n=1}^{N} |\boldsymbol{X}_n^{(i)}|^2}$

(4)

where $\boldsymbol{X}_n^{(i)}$ is the net error between the scattered and local electric field vectors in Eq. (1),

$$\boldsymbol{X}_n^{(i)} = \boldsymbol{E}_{inc,n} + \boldsymbol{E}_{scatsum,n}^{(i)} - \boldsymbol{E}_{loc,n}^{(i)}, \quad (5)$$

and $\boldsymbol{Y}_n^{(i)}$ tracks the net error between these two fields and the error propagated from every other dipole,

$$\boldsymbol{Y}_n^{(i)} = -\boldsymbol{X}_n^{(i)} + \sum_{\substack{m=1 \\ m\neq n}}^{N} \boldsymbol{G_{nm}} \cdot \boldsymbol{X}_m^{(i)}. \quad (6)$$

Convergence is achieved when both $\boldsymbol{X}_n^{(i)}$ and $\boldsymbol{Y}_n^{(i)}$ approach zero with each iterative step. For dielectric and/or isotropic material, it is usually true that the error propagated from every other dipole is smaller than the local error at the nth dipole. Thus the condition for $\boldsymbol{X}_n^{(i)} \to 0$ and $\boldsymbol{Y}_n^{(i)} \to 0$ is usually satisfied as we follow the optimization scheme Eq. (4) along each iteration step i. Then, as $g^{(i)} \to 0$, the CSDDA algorithm settles into a converged solution (i.e. $R^{(i+1)} \to 0$).

However, for anisotropic media, a new situation arises: we can still get $g^{(i)} \to 0$ but the relative error $R^{(i+1)}$ does not converge to zero. It can even be as high as 100 %. The reason for this paradox is the anisotropy of the medium, which allows for the following situation to emerge:

$$\sum_{n=1}^{N} \boldsymbol{X}_n^{(i)*} \cdot \boldsymbol{Y}_n^{(i)} \to 0\,. \tag{7}$$

Thus, from Eq. (4), we have still $g^{(i)} \to 0$, but there is no guarantee for the relative error $R^{(i+1)} \to 0$, and in fact it does not (Fig. *CSDDA-1*).

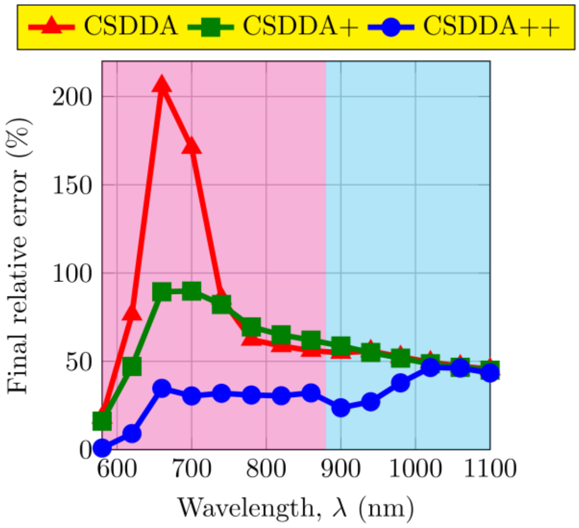

Fig. *CSDDA-1* Comparison of relative error around $g^{(i)} \to 0$, when the iteration scheme practically stagnates, for anisotropic CuSe bulk media: conventional CSDDA (red triangle), double complex CSDDA (CSDDA+) without taking into account error propagation effects (green squares), and CSDDA++ that takes into account both the error propagation and allows optimization over largest complex parameter space (blue circles). We clearly see the massive errors in conventional CSDDA approach for anisotropic media, especially in the hyperbolic domain, where the anisotropy is the most pronounced. The hyperbolic regime therefore needs the full CSDDA++ iteration scheme shown in Eq. (9). The errors in the metallic domain are primarily due to the high field enhancement at the tips of the triangle.

Expanding Eq. (7), we can understand the source of the large errors:

$$-|\boldsymbol{X}_n^{(i)}|^2 + \sum_{\substack{m=1 \\ m\neq n}}^{N} \boldsymbol{X}_n^{(i)*} \cdot \boldsymbol{G}_{nm} \cdot \boldsymbol{X}_m^{(i)} \to 0 \Rightarrow |\boldsymbol{X}_n^{(i)}|^2 \simeq \sum_{\substack{m=1 \\ m\neq n}}^{N} \boldsymbol{X}_n^{(i)*} \cdot \boldsymbol{G}_{nm} \cdot \boldsymbol{X}_m^{(i)} \tag{8}$$

or, in other words, the error propagated to dipole n from every other dipole is on the same order of magnitude as the local error at dipole n. This systemic error accumulation in CSDDA thus needs to be countered.

This has to be done in two ways:

1. by acknowledging the impact of error propagating from every other dipole $m \neq n$, in the iteration scheme Eq. (1) itself, and
2. by allowing for an optimization scheme over a much larger complex parameter space than in Eq. (1).

Not including the error propagation effects while still expanding the complex parameter space (using two optimizing hyperparameters $g^{(i)}$ and $h^{(i)}$ in Eq. (1) instead than just one) is still not sufficient, cf.~the date shown with green squares vs. those in blue circles in Fig. *CSDDA-1*.

We thus use the updated CSDDA method, which we call CSDDA++ (expanding to second-order propagation and expanding the optimizing parameter space), to implement the generalized recursive iteration scheme,

$$\boldsymbol{E}_{loc,n}^{(i+1)} = \alpha^{(i)}\left(\boldsymbol{E}_{inc,n} + \boldsymbol{E}_{scatsum,n}^{(i)}\right) + \beta^{(i)}\boldsymbol{E}_{loc,n}^{(i)} + \gamma^{(i)} \sum_{\substack{m=1 \\ m\neq n}}^{N} \boldsymbol{G}_{nm} \cdot \left(\boldsymbol{E}_{inc,m} + \boldsymbol{E}_{scatsum,m}^{(i)}\right) + \delta^{(i)} \sum_{\substack{m=1 \\ m\neq n}}^{N} \boldsymbol{G}_{nm} \cdot \boldsymbol{E}_{loc,m}^{(i)} \tag{9}$$

which allows for more consistent and better convergence. The optimization parameters $\alpha^{(i)}$, $\beta^{(i)}$, $\gamma^{(i)}$, and $\delta^{(i)}$ are calculated for each iteration step i using the same principle as in the original CSDDA, i.e. by minimizing the upgraded relative error $R^{(i+1)}$,

$$R^{(i+1)} = \sqrt{\frac{\sum_{n=1}^{N} \left|\boldsymbol{E}_{inc,n} + \boldsymbol{E}_{scatsum,n}^{(i+1)} + \sum_{\substack{m=1 \\ m\neq n}}^{N} \boldsymbol{G}_{nm} \cdot \left(\boldsymbol{E}_{inc,m} + \boldsymbol{E}_{scatsum,m}^{(i+1)}\right) - \boldsymbol{E}_{loc,n}^{(i+1)} - \sum_{\substack{m=1 \\ m\neq n}}^{N} \boldsymbol{G}_{nm} \cdot \boldsymbol{E}_{loc,m}^{(i+1)}\right|^2}{N}} . \tag{10}$$

We thus obtain a linear algebraic equation in the 4 unknowns $\alpha^{(i)}$, $\beta^{(i)}$, $\gamma^{(i)}$, and $\delta^{(i)}$ for each iteration step i, which then updates the local electeric field at each dipole n.

Finally, we also include the impact of the substrate on which the nanocrystals are grown. This can be approximated with good accuracy through the Green's function of an infinite plate (the NC is placed just one dipole thickness above the plate). Then for each dipole n, we have a scattering term $\boldsymbol{G}_{nn}$ that contributes to the iterative scheme so that $m = n$ contributions should also be included,

$$\boldsymbol{E}_{loc,n}^{(i+1)} = \alpha^{(i)}\left(\boldsymbol{E}_{inc,n} + \boldsymbol{E}_{scatsum,n}^{(i)}\right) + \beta^{(i)}\boldsymbol{E}_{loc,n}^{(i)} + \gamma^{(i)} \sum_{m=1}^{N} \boldsymbol{G}_{nm} \cdot \left(\boldsymbol{E}_{inc,m} + \boldsymbol{E}_{scatsum,m}^{(i)}\right) + \delta^{(i)} \sum_{m=1}^{N} \boldsymbol{G}_{nm} \cdot \boldsymbol{E}_{loc,m}^{(i)} . \tag{11}$$

Note that the term $m = n$ is also included in the summations. The Green's function has the following expression depending on values of m and n relative to each other[13–15],

$$\boldsymbol{G}_{nm} \cong \boldsymbol{G}(\boldsymbol{r}_n, \boldsymbol{r}_m, \omega) = \frac{e^{ik\rho}}{4\pi\varepsilon_0\rho^3}[-\{1 - k\rho - (k\rho)^2\}\boldsymbol{I} + \{3 - 3ik\rho - (k\rho)^2\}\frac{\boldsymbol{\rho}\otimes\boldsymbol{\rho}}{\rho^2}], m \neq n, \quad (12)$$

$$\boldsymbol{G}_{nn} \cong \boldsymbol{G}(\boldsymbol{r}_n, \boldsymbol{r}_n, \omega) = \frac{1}{32\pi\varepsilon_0 z_n^3}\frac{\varepsilon(\omega)-1}{\varepsilon(\omega)+1}\begin{pmatrix} 1 & 0 & 0 \\ 0 & 1 & 0 \\ 0 & 0 & 2 \end{pmatrix}, \quad m = n \quad (13)$$

Due to the strong absorption in most of the spectral domain, it is a reasonable approximation to consider the Green's functions in the near-field limit $(k\rho) \ll 1$. The final results do not differ from calculations for the full Green's function, but due to limited propagation effects, offer faster convergence at the same time. Also, in Gaussian units, $\varepsilon_0 = 1/4\pi$.

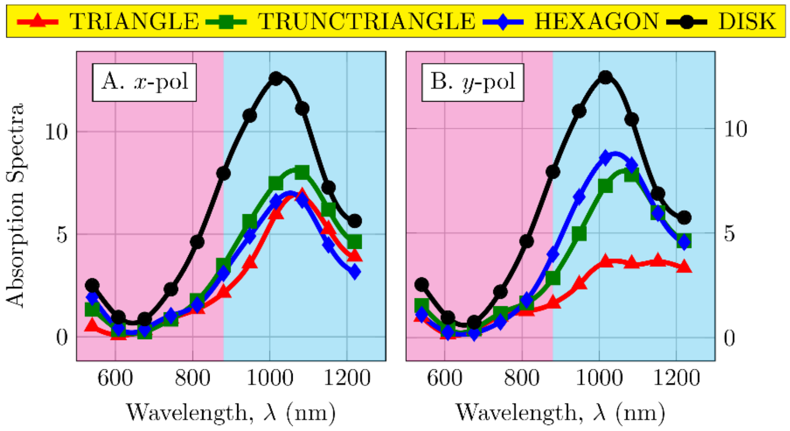

Fig. S6 Intensity-normalized absorption spectra for CuSe NCs of different shapes, with same diagonal length *a* = 19 nm, and thickness *c* = 5 nm, for incident field polarized along the (A) *x*-axis and (B) *y*-axis, $E_{inc} = e^{ikz}$ (total number of dipoles, $N_d = 80 \times 80 \times 20 = 128{,}000$).

CSDDA simulations for different angles of incidence

By changing the angle of incidence from $\theta_k = 0$ to $\theta_k = 60$ degrees, we can gradually increase the LSPR absorption cross-section in the hyperbolic spectral range (Fig. S7 and Fig. S8). At $\theta_k = 75$ degrees, the hyperbolic LSPR dominates entirely over the metallic LSPR – we observe this effect across all crystal shapes. The disk NC (shown here for comparison purpose), due to its high symmetry, has nearly identical absorption spectra in Fig. 8 and 9, for a given incidence angle θ_k (the minor discrepancy for $\theta_k = 45$ degrees are likely due to DDA errors).

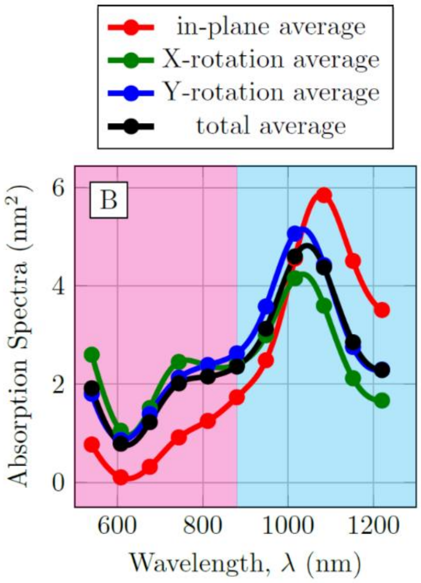

Fig. S7 Rotational averaging of the absorption spectra for changing polarization in-plane of the NC surface, from CSDDA++. The hyperbolic domain is shown in pink.

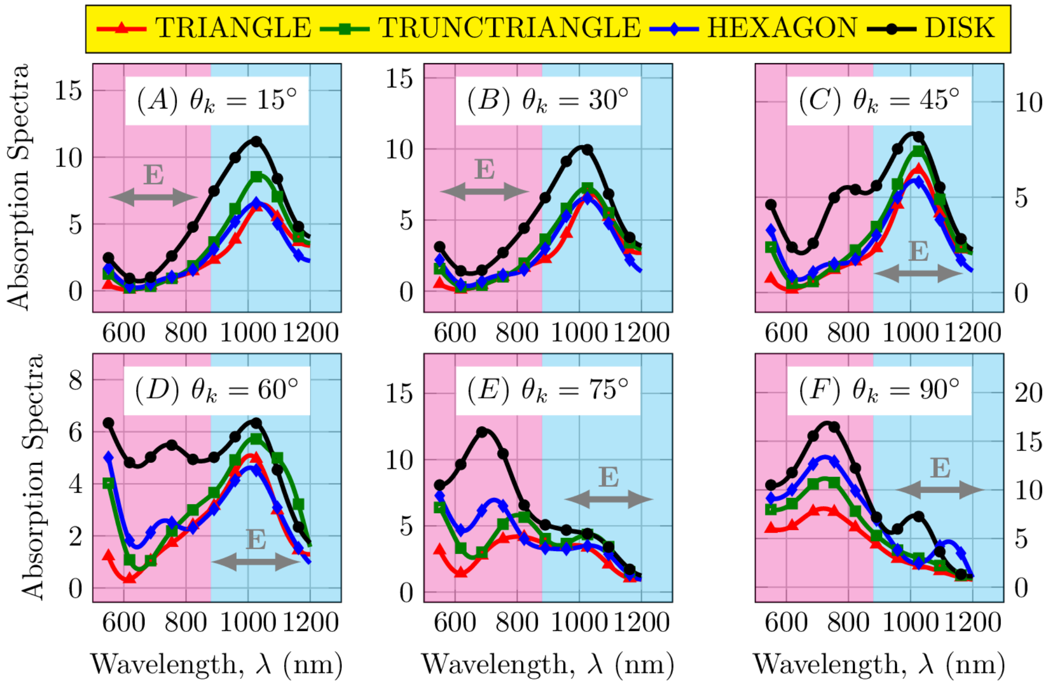

Fig. S8 Intensity-normalized absorption cross-section for triangle (red triangles), truncated triangle (hexatrian) (green squares), hexagon (blue diamond), and disk (magenta circles) NC geometry, with total number of dipoles, $N_d = 80 \times 80 \times 20 = 128{,}000$. The incident field is polarized along the *x*-axis, with angle of incidence θ_k (A-F). Absorption cross-section can be enhanced to as high as 17 nm^2 (F), by controlling the angle of incidence. Gradually increasing the incidence angle also amplifies the LSPR in the hyperbolic regime, while suppressing the LSPR in the metallic domain. By $\theta_k = 90$ degrees (F), the plasmon resonances look qualitatively identical for every pr shape.

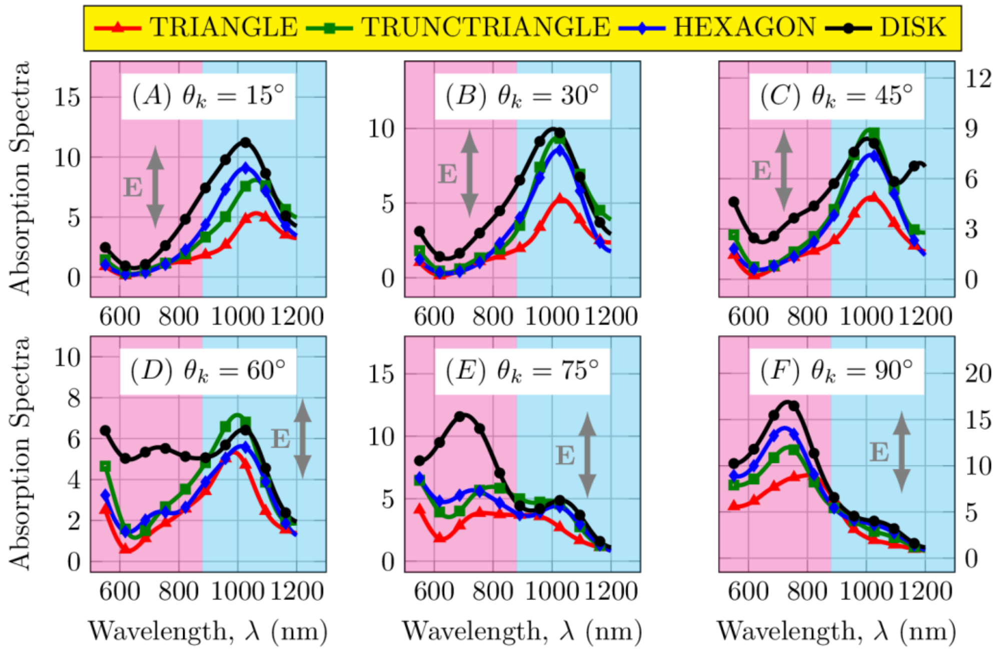

Fig. S9 Intensity-normalized absorption cross-section for triangle (red triangles), truncated triangle (hexatrian) (green squares), hexagon (blue diamond), and disk (magenta circles) NC geometry, with total number of dipoles, $N_d = 80 \times 80 \times 20 = 128{,}000$. The incident field is polarized along the *y*-axis, with angle of incidence θ_k (A-F). Absorption cross-section can be enhanced to as high as 15 nm^2 (F), by controlling the angle of incidence. Gradually increasing the incidence angle also amplifies the LSPR in the hyperbolic regime, while suppressing the LSPR in the metallic domain By $\theta_k = 90$ degrees (F), the plasmons look almost identical for every crystal shape.

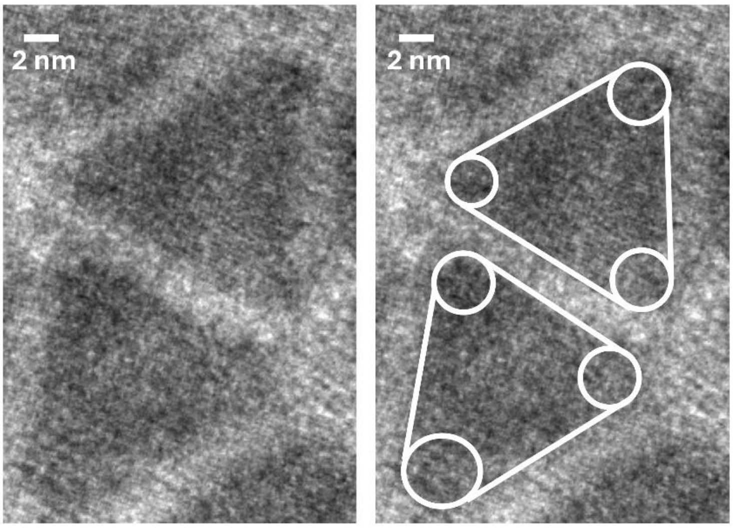

Fig. S10 TEM image of triangular CuSe nanoprisms without (left) and with (right) alignment of the vertices with overlayed radial curvatures.

The impact of the intrinsic material anisotropy can be deciphered, by comparing our field distribution maps in Fig. 11, to the corresponding case of *isotropic* triangular nanoprism in Figs. 12a and 12b.

First, we assume a triangular nanoprism with the same shape and dimensions as in Fig. S11, but with isotropic permittivity ε_{xx}, which remains metallic throughout the spectral range of interest. As seen in Fig. S12a, we obtain the standard metallic response with strong dipolar and quadrupolar LSPRs and high amplification. However, at no wavelength do we observe the "enveloping" mode that appears at λ = 773 nm in the anisotropic case. Similarly, we consider an isotropic triangular nanoprism with permittivity ε_{zz} applied both in-plane and out-of-plane (Fig. S12b). Here, the optical response gradually shifts from dielectric to metallic. In the dielectric regime, the plasmonic excitations tend to "leak" into the environment, and even at λ = 773 nm the isotropic nanoprism remains weakly dielectric. As the wavelength enters the NIR range, the ε_{zz}-nanoprism becomes metallic, but only edge-confined quadrupolar excitations appear, with gradual extinction of the plasmonic response. In contrast, the anisotropic hyperbolic nanoprism (Fig. S11b) supports strongly confined excitations and clearly exhibits the full-surface "enveloping" mode. Neither isotropic case reproduces this effect or the considerably stronger enhancement seen in the anisotropic structure, which results from its in-plane metallic excitations. We thus see direct evidence that the enveloping modes are a direct consequence of intrinsic material anisotropy.

In conclusion, shape anisotropy is responsible for blue shifts, hotspots formation, asymmetric spectral broadening (as one moves from disk to hexagon to truncated triangle to triangle geometry in Figs. S8 and 9 for any incidence angle θ_k), while the intrinsic material anisotropy is directly responsible for enveloping modes shown close to epsilon near zero (ENZ) wavelengths in a naturally hyperbolic material.

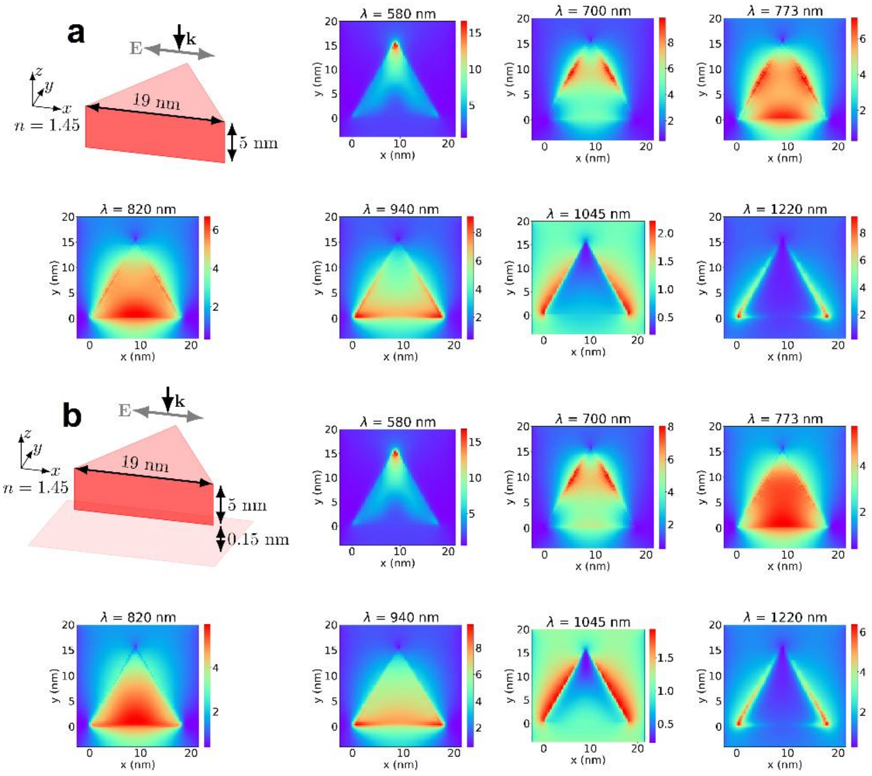

Fig. S11 Substrate effect on the E-field distribution in triangular CuSe nanocrystal (CSDDA simulation). (a) NC in a solvent without the substrate (repeated for comparison). (b) NC in a solvent on a substrate.

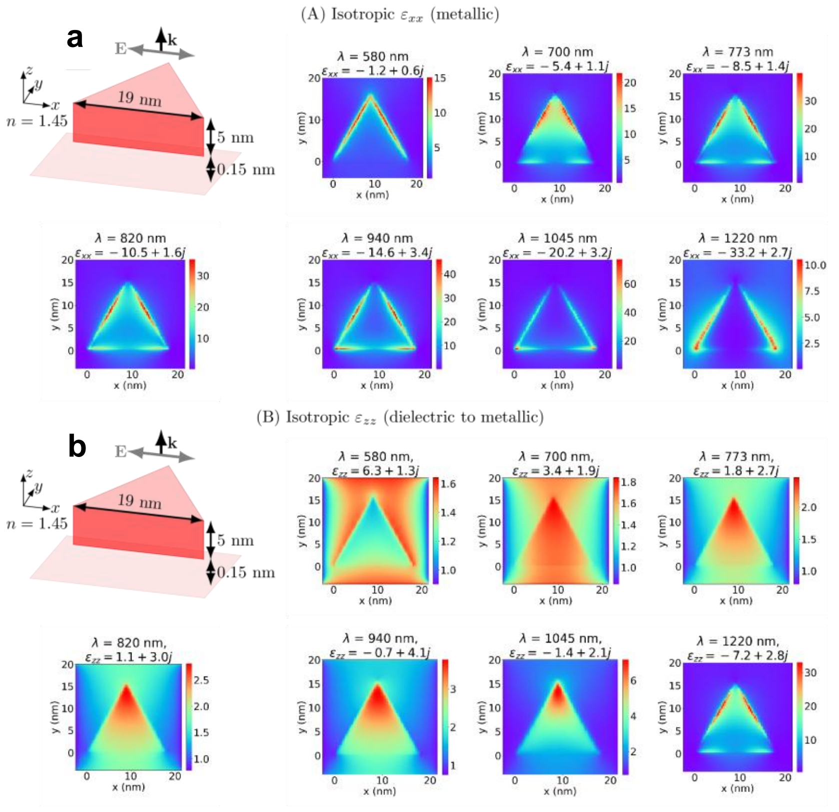

Fig. S12 Intensity-field profile around an “model” triangular nanocrystal with isotropic permittivity for (a) ε_{xx}-isotropy and (b) ε_{zz}-isotropy. Comparing with Fig S11b, we can see the impact of the material anisotropy.

4. Fitting of TA oscillatory features

The averaged time traces were fitted using a model consisting of two exponential decay terms and an oscillatory component:

$$y = A_1 \cdot e^{\frac{-t}{\tau_1}} + A_2 \cdot e^{\frac{-t}{\tau_2}} + B \cdot e^{\frac{-t}{\tau_d}} \cdot \cos\left((2\pi f \cdot t) + \varphi\right)$$

where the amplitudes A_1, A_2 and the time constants τ_1 und τ_2 describe the exponential decay components, while B, τ_d, f and φ represent the amplitude, damping constant, frequency and phase of the oscillation, respectively. The relevant extracted values are summarized in Table 1 of the main text. The oscillation frequency obtained from the fit is 7.5 THz for NSs and 7.8 THz for NPLs, which is in good agreement with the FFT results.

5. Fourier Transform of TA data for different spectral regions

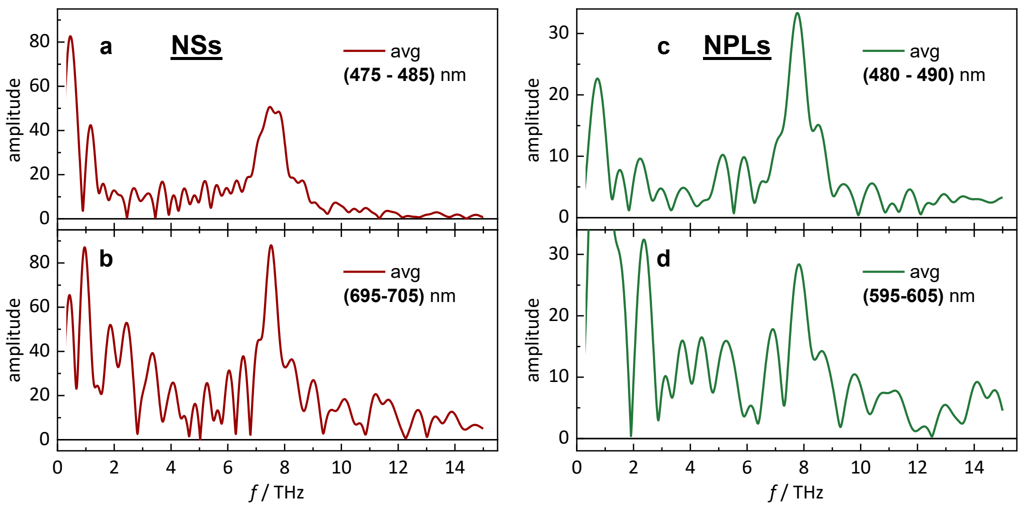

Fig. S13 (a) and (c) FFT data of the NSs and NPLs averaged over a spectral window in the flank of the UV absorption. **(b)** and **(d)** FFT data of the NSs and NPLs averaged over a spectral window at the absorption minimum of the stationary absorption spectra. For both types of particles, the Fourier amplitudes of the oscillations in the TA signal are similar in both spectral windows.

References

(1) Kraus, W.; Nolze, G. POWDER CELL–a program for the representation and manipulation of crystal structures and calculation of the resulting X-ray powder patterns. *Applied Crystallography* **1996**, *29*, 301–303.

(2) Córdova-Castro, R. M.; Casavola, M.; van Schilfgaarde, M.; Krasavin, A. V.; Green, M. A.; Richards, D.; Zayats, A. V. Anisotropic plasmonic CuS nanocrystals as a natural electronic material with hyperbolic optical dispersion. *ACS Nano* **2019**, *13*, 6550–6560.

(3) Thomas, S.; Hildreth, O.; Zaeem, M. A. Unveiling the role of atomic defects on the electronic, mechanical and elemental diffusion properties in CuS. *Scripta Materialia* **2021**, *192*, 94–99.

(4) Mazin, II. Structural and electronic properties of the two-dimensional superconductor CuS with 1 1 3-valent copper. *Physical Review B—Condensed Matter and Materials Physics* **2012**, *85*, 115133.

(5) Gaspari, R.; Manna, L.; Cavalli, A. A theoretical investigation of the (0001) covellite surfaces. *The Journal of Chemical Physics* **2014**, *141*.

(6) Paliwal, S. S.; Maurya, V.; Joshi, K. B. First-principles study of electronic structure and fermiology of covellite mineral and its B1, B3 phases. *Journal of Physics: Condensed Matter* **2020**, *32*, 295501.

(7) Hedin, L. On correlation effects in electron spectroscopies and the GW approximation. *Journal of Physics: Condensed Matter* **1999**, *11*, R489.

(8) Hedin, L. New method for calculating the one-particle Green's function with application to the electron-gas problem. *Physical Review* **1965**, *139*, A796.

(9) Reining, L. The GW approximation: content, successes and limitations. *Wiley Interdisciplinary Reviews: Computational Molecular Science* **2018**, *8*, e1344.

(10) Aryasetiawan, F.; Gunnarsson, O. The GW method. *Reports on progress in Physics* **1998**, *61*, 237.

(11) J. J. De Yoreo. Principles of Crystal Nucleation and Growth. *Reviews in Mineralogy and Geochemistry* **2005**, *54*, 57–93.

(12) Sander, K.; Peltz, C.; Varin, C.; Scheel, S.; Brabec, T.; Fennel, T. Influence of wavelength and pulse duration on single-shot x-ray diffraction patterns from nonspherical nanoparticles. *Journal of Physics B: Atomic, Molecular and Optical Physics* **2015**, *48*, 204004.

(13) Tai, C.-T. Dyadic Green's functions in electromagnetic theory. *(No Title)* **1971**.

(14) Scheel, S.; Buhmann, S. Y. Macroscopic quantum electrodynamics-concepts and applications. *Acta Phys. Slovaca* **2008**, *58*, 675–809.

(15) Buhmann, S. Y. *Dispersion Forces I: Macroscopic quantum electrodynamics and ground-state Casimir, Casimir–Polder and van der Waals forces*; Springer, 2013.
